# Nighttime lights as a proxy for human development at the local level

**DOI:** 10.1371/journal.pone.0202231

**Published:** 2018-09-05

**Authors:** Anna Bruederle, Roland Hodler

**Affiliations:** Department of Economics and SIAW-HSG, University of St.Gallen, St.Gallen, Switzerland; Universitat Jaume I, SPAIN

## Abstract

Nighttime lights, calculated from weather satellite recordings, are increasingly used by social scientists as a proxy for economic activity or economic development in subnational regions of developing countries where disaggregated data from statistical offices are not available. However, so far, our understanding of what nighttime lights capture in these countries is limited. We use geo-referenced Demographic and Health Surveys (DHS) from 29 African countries to construct indicators of household wealth, education and health for DHS cluster locations as well as for grid cells of roughly 50 × 50 km. We show that nighttime lights are positively associated with these location-specific indicators of human development, and that the variation in nighttime lights can explain a substantial share in the variation in these indicators. We conclude that nighttime lights are a good proxy for human development at the local level.

## Introduction

Economic and social data for subnational administrative regions, such as provinces, districts or municipalities, are unavailable for most developing countries, and of poor quality if they exist. For geographical units that cut across administrative borders, such as ethnic homelands, ecological zones or grid cells, reliable economic and social indicators are even rarer. This lack of spatially disaggregated data has inhibited empirical research on important questions in economics and related social sciences for many years. In the absence of (reliable) subnational data, social scientists have recently resorted to an alternative measure that does not depend on data collection on the ground: nighttime lights calculated from weather satellite recordings and made available by the U.S. National Oceanic and Atmospheric Administration (NOAA) as an annual time series. Key benefits of these data are the global coverage and the high spatial resolution with pixels corresponding to less than one square kilometer, which allows researchers to aggregate these data at the level of the subnational units they want to study. In addition, nighttime lights are measured with consistent quality across countries with very different institutional capacities, and are not susceptible to politically motivated manipulation.

Following a series of studies that proposed nighttime lights as a proxy for economic outcomes [1–8], nighttime lights data have been used to measure economic activity or economic development within administrative regions [9, 10], cities and municipalities [11, 12], ethnic homelands [13–16], and grid cells of various sizes [7, 12, 17–19]. Thereby, nighttime lights have helped to improve our understanding of comparative development [13, 14, 17, 18] as well as a host of other topics ranging from favoritism [9, 16] to ethnic inequality [15] and micro-finance [11].

We expect that the use of nighttime lights in the social sciences will continue to grow. First, many more social scientists are acquiring the skill set necessary to work with spatial data. Second, nighttime lights have been added to the PRIO-GRID data set [20], which provides time series of a rich set of variables in a standardized spatial grid structure with cells of 0.5 × 0.5 decimal degrees (corresponding to approximately 55 × 55 km at the equator).

The surge in the use of nighttime lights contrasts with our rather limited understanding of what nighttime lights do and do not capture. We see at least two important gaps in the evidence that underpins the validity of nighttime lights as a tool in the social sciences. First, we do not know whether nighttime lights are an accurate proxy for economic activity and economic development for small spatial units such as municipalities in developing countries. Second, we do not know whether nighttime lights also capture other important dimensions of human development such as education and health.

We aim to fill these gaps. We explore the relationship between nighttime lights and human development at the local level in Africa. We focus on Africa, where the lack of disaggregated data to measure human development is particularly eminent. We use geo-referenced data from the Demographic and Health Surveys (DHS) to construct local measures of household wealth, education and health, and relate them to nighttime lights. We use two different spatial units for our analysis, differing in their resolution: circular zones with a radius of up to 5 km around DHS cluster locations, and PRIO-GRID cells.

We find that more intense nighttime lights are associated with better human development outcomes in terms of household wealth, education and health. For example, around 50 percent of the within-country variation in household wealth, school attendance, years of education, and the share of births assisted by professional health personnel is explained by variation in nighttime lights. This association holds both across our small circular zones and across the larger PRIO-GRID cells. We also document that nighttime lights contain information about variation in local human development after controlling for local population density, urbanization and electrification. We conclude that nighttime lights are a good proxy not only for local economic development, but also for other dimensions of local human development related to education and health.

Our study contributes to the literature on the relation between nighttime lights and purely economic outcomes. There is evidence for a positive relation of nighttime lights with gross domestic product (GDP) at the level of countries [8], provinces [9], and relatively large grid cells of 1 × 1 decimal degrees [7, 21]. These latter studies find that nighttime lights can mainly improve estimates of economic output in countries with poor statistical systems [7, 21]. This finding is consistent with our reason for focusing on Africa, where many countries have poor statistical systems. A positive relation between nighttime lights and a survey-based wealth index has been documented at the level of provinces [4], and nighttime lights have also been shown to be a good predictor of local wealth as measured by the DHS wealth index [22]. Another recent study estimates consumption and wealth at the level of survey clusters relying on a machine learning technique that processes daytime satellite images and nighttime lights [23]. The most fine-grained analysis of how nighttime lights are associated with economic outcomes is provided in a study on Sweden [24]. This study uses geo-referenced data on population, enterprises, employment and wages at a resolution of 250 × 250 meter grid cells in urban and 1 × 1 km grid cells in rural areas. Data at this level of spatial accuracy are however typically not available for developing countries, which is one reason why nighttime lights are arguably most promising for these countries. None of these studies has looked at the relation between nighttime lights and economic outcomes in developing countries across PRIO-GRID cells or similarly sized spatial units. In addition, none of the studies that include developing countries has explicitly studied whether nighttime lights contain useful information on local economic activity beyond population density, urbanization, and electrification. We do by controlling for these variables in our estimates and also by constructing an electricity-independent wealth measure.

More importantly, we contribute to the nascent literature on the relation between nighttime lights and human development more broadly defined. Nighttime lights have been found to be correlated with infant mortality and poverty rates at the level of provinces [25], but we are not aware of any study that looks at various human development outcomes, e.g., related to education, and at a higher spatial resolution. This may be surprising given that the question about the relation between nighttime lights and human development is reminiscent of the old debate on whether GDP per capita is a proxy not only for economic development narrowly defined, but also for human development more broadly defined (see [26] for a recent contribution to this old debate).

## Materials and methods

### Data

We use the nighttime lights data by the National Oceanic and Atmospheric Administration (NOAA) and combine them with 71 geo-coded Demographic and Health Surveys (DHS) from 29 African countries for the years 1992 to 2013. Before discussing these data in detail, we introduce the spatial units that we use in our analysis. First, we use circular zones around reported center points of DHS clusters, with a 2 km radius for urban clusters and a 5 km radius for rural clusters. Second, we use grid cells of 0.5 × 0.5 decimal degrees, aligned to the PRIO-GRID [20]. Our samples comprise around 29,000 circular zones and 7,500 PRIO-GRID cells.

**Fig 1:**
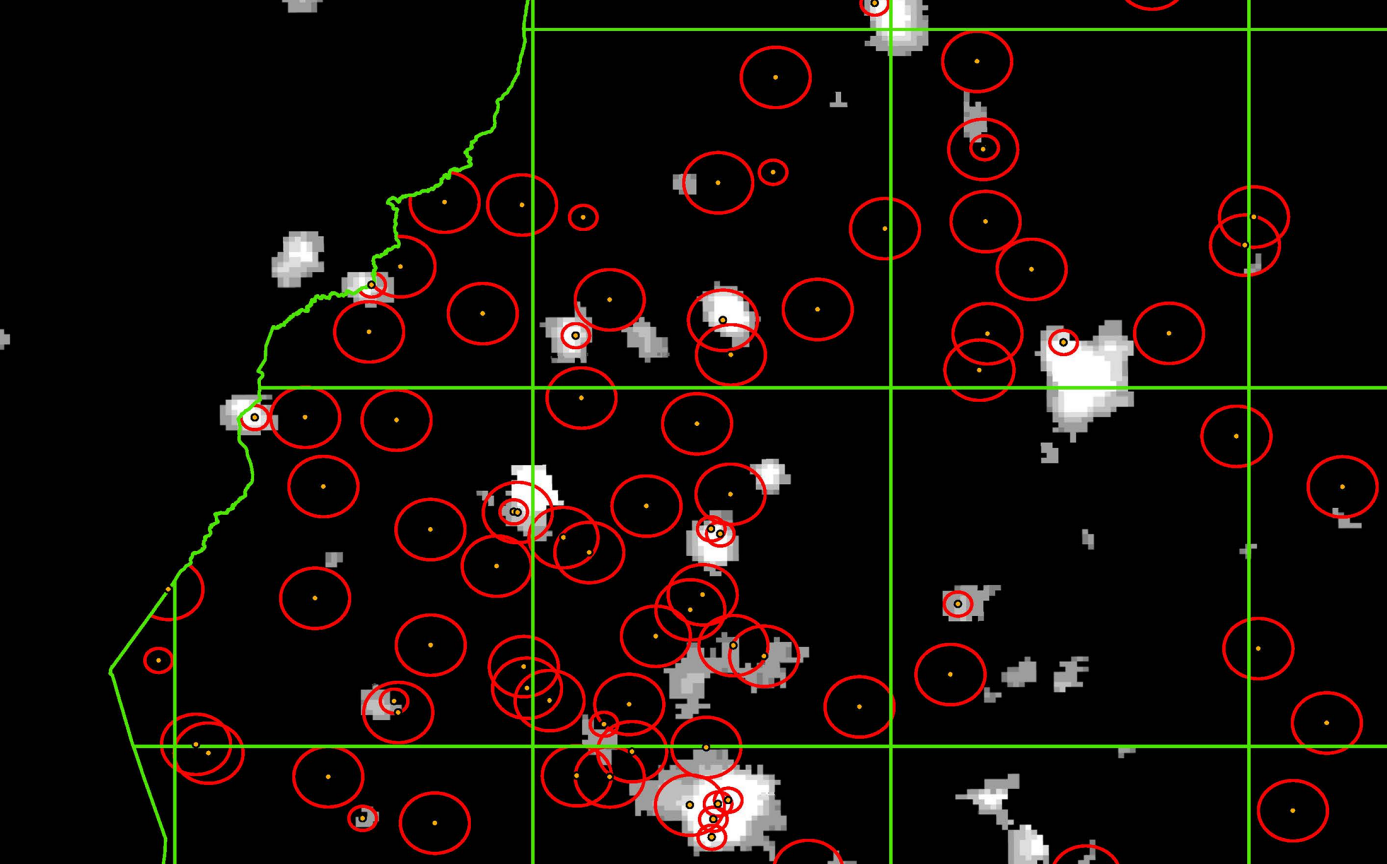
Illustration of DHS clusters, nighttime lights and our spatial units for Western Kenya. Dots represent reported DHS cluster center points from Kenya DHS 2008. Circular zones around these center points (in red) have a 2 km radius for urban clusters and a 5 km radius for rural clusters. The grid cells of 0.5 × 0.5 decimal degrees (in green) are aligned to the PRIO-GRID. Nighttime lights underlay the map, with brighter tones of grey implying more intense nighttime lights.

Fig 1 illustrates these two different spatial units for Western Kenya. The dots represent the reported DHS cluster center points and the (red) circles around these dots our circular zones. The (green) rectangular cells are the PRIO-GRID cells. These spatial units are clipped at country borders (as can be seen on the left in Fig 1) and coastal lines. Brighter tones of grey in the background indicate more intense nighttime lights.

We study the association between nighttime lights and local human development at the level of both small circular zones and PRIO-GRID cells for two reasons. First, we would like our study to inform research exploiting the high spatial resolution of the nighttime lights data as well as research based on the PRIO-GRID. Second, presenting results for two different types of spatial units helps to address the modifiable areal unit problem.

#### Nighttime lights as the main explanatory variable

Our nighttime lights variable is based on satellite images collected by the Operational Linescan System sensors installed on satellites of the Defense Meteorological Satellite Program. These weather satellites circle the earth several times per day and collect a digital stream of images relevant for weather observation and forecasting. The sensors are designed to help identify cloud coverage at night through detecting moonlight reflections, but on cloud-free nights they record light emissions from the earth’s surface. These images are processed by the NOAA into global annual composites of cloud-free nighttime lights.

The NOAA currently provides annual data for the time period from 1992 to 2013 [27]. Following most of the economic literature, we choose the Stable Lights series from among the NOAA’s various nighttime light products for our analysis. This is a series of global maps showing the relative nighttime light intensities on the earth’s surface averaged over calendar years, where transient lights that are deemed ephemeral have been filtered out and non-lit areas are set to zero. The steps of screening and filtering of the raw images, both manual and by automated algorithms, made to obtain the Stable Lights are described in [28]. The data come in gridded format with pixels of 30 × 30 arc seconds. This pixel size corresponds to less than one square kilometer at the equator. For each of the pixels, annual average light intensity is reported in digital numbers (DN) ranging from 0 to 63, with higher values implying more intense nighttime light.

For the majority of years, Stable Lights are available from two different satellites. For these years, we use the data from the newer satellites in our main analysis. We show in S2 Table that our results are very similar when using data from the older satellite for each of these years.

We construct from these data our nighttime lights variable (labelled light) as the average DN of all nighttime lights pixels within a spatial unit for the year in which the respective DHS survey was carried out (or for the earlier year if DHS data collection extended over two calendar years). These spatial units are our small circular zones and the PRIO-GRID cells, whereby the former nicely illustrate how the high spatial resolution of the nighttime lights data allow studying the very local level.

An alternative nighttime lights variable (labelled sum of light) corresponds to the sum of the DN of all nighttime lights pixels in a spatial unit. This variable tends to have higher values for spatial units with higher average DN or larger area. From the perspective of researchers looking for a proxy for human development, the bias in favor of larger spatial units is typically undesirable. This bias may be substantial for spatial units such as administrative regions or ethnic homelands, which typically vary strongly in size. The spatial units we analyse vary in size as well, because we clip them at country borders and coastal lines. For this reason, we choose the average DN of nighttime lights for our main analysis, but present robustness tests using the sum of light in S3 Table.

The Stable Lights series has two caveats that can be a concern for applications in the social sciences. First, DN are often top-coded at 63 in the centers of metropolitan areas due to calibration of the sensors which allows to detect very low levels of illuminance. The Stable Lights maps hence do not allow to distinguish between bright urban centers and their periphery. However, for the African continent the fraction of pixels in the original data with DN 63 is less than 0.06 percent, which is why we do not consider it a major concern for most studies focusing on Africa.

The second caveat is that many pixels have DN 0. In our sample, 51 percent of the circular zones around reported DHS cluster center points and 42 percent of the PRIO-GRID cells have zero average nighttime light, as they contain only pixels with DN 0. Looking at how these shares vary across countries and over time provides three insights (see S1 Fig): First, the cross-country variation in these shares is considerably larger than the variation within countries over time. Second, the share of spatial units with zero average nighttime light has increased over time in our sample. Third, this share has decreased over time for most countries with multiple DHS. Hence, the overall increase in the share of spatial units with zero average nighttime light in our sample is mainly the result of compositional changes over time. For example, Egypt has almost no areas with zero average nighttime light (nearby DHS cluster locations) and conducted comparatively many DHS in the early years of our sample period but few in later years. Since we know that all spatial units in our sample are inhabited, we assume that even spatial units with zero average nighttime light are not completely dark, but that their non-zero light emissions could not be recorded by the weather satellites or were filtered away in the preparation of the Stable Lights series.

#### DHS-based development indicators as dependent variables

Our dependent variables are constructed from the Demographic and Health Surveys (DHS), which are large periodic household surveys that have been carried out in low-income countries in Africa and elsewhere since the 1980s [29]. These surveys primarily collect information from women at childbearing age on a wide range of topics related to health, nutrition, fertility and education, as well as a set of household characteristics such as access to infrastructure and ownership of household assets. In each country, households are selected to produce nationally representative samples. Usually, sampling is done through a stratified cluster design, based on the country’s most recent population census, in two stages. At the first stage, clusters are drawn from official listings of census enumeration areas, which in most countries correspond to small villages or blocks within larger villages or cities. At the second stage, a sample of households is drawn randomly from a list of households in each cluster. Mean outcomes for a cluster should thus provide an accurate measure of the local level of development.

For those DHS that are geo-referenced, the data contain geo-coordinates of the cluster center points, usually recorded with Global Positioning System (GPS) receivers. The actual location of each household is not recorded, and the DHS provide no indication how far households are scattered around these cluster center points. To further ensure confidentiality of the respondents, some noise is added to the coordinates by displacing each cluster center point in a random direction and by a random distance of 0–2 km for urban clusters, and 0–5 km for rural clusters, with 1 percent of rural clusters displaced by up to 10 km. This displacement is the reason why we choose circular zones with a radius of 2 km for urban clusters and 5 km for rural cluster as the high spatial resolution units in our analysis.

Our sample includes all 71 survey waves with GPS-measured geo-coordinates that have been carried out in African countries over the time period for which nighttime lights data are currently available, i.e., 1992–2013. These survey waves were conducted across 29 different countries from all over Africa (see S1 Table). DHS follow a largely consistent methodology and structure across countries and years, which is why they qualify for an analysis of local development in a sample with many countries and survey waves.

In order to measure human development in our sample localities, we look at the three key dimensions of human development as reflected in the Human Development Index: a decent standard of living, education, and health. For each of these dimensions, we construct two indicators for which the DHS in our sample provide the relevant data.

We measure standard of living based on household wealth, partly because DHS do not collect data on household income or consumption. As our main wealth measure, we use the DHS wealth index whenever it is available. The DHS wealth index measures households’ relative economic status by categorizing them into five strata. These strata are based on quintiles on the distribution of a wealth score, which is computed as a linear combination of a set of wealth indicators, including whether the household owns selected assets; the type of water and sanitation facilities; access to electricity; and the housing quality. For each survey, the weights of these indivial wealth indicators are derived by principal component analysis (see [30] for a detailed description of the DHS wealth index). A wealth index value of one is assigned to the poorest fifth, and five is assigned to the wealthiest fifth of households within the survey wave. For surveys for which the DHS wealth index is not available, we compute an analogous index following the DHS methodology based on the wealth indicators that are available across all surveys.

Electricity access is one component of the DHS wealth index, and several components are household assets that depend on electricity access for their use. Yet electricity access is inherently correlated with nighttime lights. We therefore construct a second wealth index (labelled e-free wealth), where we include only indicators that do not depend on electricity and are available across all surveys (i.e., ownership of bicycle, motorcycle, and car; floor materials; type of drinking water source; and type of toilet facility). We thereby again follow the DHS methodology, such that this second wealth index also various from one to five. Note that since these two wealth indices measure households’ relative wealth within the survey wave, they cannot be used for comparisons of wealth across countries and surveys.

We measure education using primary school attendance and the number of years of schooling. One can interpret the former as a flow and the latter as a stock measure of education. We calculate the net attendance ratio in primary education (labelled school attendance) as the ratio of the number of children of official primary school age (as defined by the national education system) who have attended primary school in the year preceding the survey to the total population of children of official primary school age. The number of years of schooling is based on all household members aged 18 or older.

We measure health using the infant mortality rate (labelled infant mortality) and the proportion of births attended by skilled health personnel (labelled birth assistance). The former is a commonly used measure of health and the latter a measure of access to health services. The infant mortality rate is defined as the number of infants that died before reaching the age of one year per 1,000 live births. In order to make full use of records for children born within a relatively short period preceding the survey, we follow the DHS methodology and derive infant mortality rates through a synthetic cohort life table approach. This approach calculates mortality probabilities for small age segments (0 months, 1-2 months, 3-5 months, 6-12 months) and combines them into the one-year age segment. We use birth records from the three years preceding the survey, because we aim to generate a relatively near-term picture of infant mortality, but require a reasonable number of births to compute the infant mortality rate. Birth assistance is the percentage of deliveries attended by a doctor, a nurse or a midwife among all births in the three years preceding the survey.

For each of these indicators, we derive shares or mean values for each single DHS cluster, which we use as the value of the dependent variables in our analysis based on circular zones around DHS clusters.

We then aggregate the human development indicators at the level of PRIO-GRID cells. We therefore intersect our 2 km or 5 km circular zones around the DHS cluster center points with the PRIO-GRID cells (using GIS). Observations from DHS clusters whose reported center point lies more than 2 km (for urban clusters) or more than 5 km (for rural clusters) from cell boundaries are entirely allocated to their respective cell. For other DHS clusters we allocate the available observations to the different nearby cells in proportion to the area share of the circular zone that falls within these cells. This procedure yields cell-level values for our development indicators for all grid cells that intersect with at least one of our circular zones. The number of observations underlying the cell-level aggregate and the number of clusters from which these observations stem vary across cells and indicators.

#### Control variables

We use three control variables. The first is population density (labelled population), which we compute based on the CIESIN Gridded Population of the World (GPWv4 and GPWv3) data sets [31]. We use the population density grids for the years 2000, 2005, 2010 and 2015 from the GPWv4, and for the years 1990 and 1995 from the GPWv3 data sets. For the years between these intervals, we construct population density estimates by linear interpolation.

The second control variable is the electrification rate (labelled electricity), which we derive from the DHS. Respondents are asked whether their household has electricity, without specifying the source. To calculate cell-level electrification rates, we use the same procedure applied to generate other cell-level development indicators.

The third control variable captures urbanization (labelled urban). DHS reports whether the clusters are urban or rural according to the classification used by the respective national statistical administration. For the small circular zones, the urban variable is simply an indicator that equals one if the DHS cluster is classified as urban, and zero if it is classified as rural. For the PRIO-GRID cells, this variable is defined as the share of urban clusters among all DHS clusters whose center point falls within the cell.

### Descriptive statistics

Table 1 provides summary statistics for all our variables for both types of spatial units used in our analysis.

**Table 1:** Summary statistics.

Variable (and unit)	Obs.	Mean	Std. Dev. overall/within	Min.	Max.
Panel A: Small circular zones					
light (DN)	29,169	11.75	19.56/14.78	0	63
household wealth (stratum)	26,759	2.953	1.147/1.068	1	5
e-free wealth (stratum)	26,702	2.869	1.161/1.076	1	5
school attendance (rate)	28,788	0.722	0.279/0.255	0	1
years of schooling	28,860	4.751	3.159/2.797	0	19
infant mortality (per 1,000)	28,818	58.91	83.74/75.37	0	1,000
birth assistance (rate)	27,901	0.565	0.349/0.315	0	1
population (per km^2^)	27,897	1,150	3,937/2,279	0	80,684
electricity (rate)	29,051	0.356	0.424/0.343	0	1
urban (rate)	29,169	0.322	0.467/0.417	0	1
Panel B: PRIO-GRID cells					
light (DN)	7,515	0.775	3.377/3.132	0	57.69
household wealth (stratum)	7,144	2.585	0.907/0.825	1	5
e-free wealth (stratum)	7,275	2.717	0.950/0.882	1	5
school attendance (rate)	7,436	0.613	0.287/0.263	0	1
years of schooling	7,442	3.796	2.657/2.363	0	12.73
infant mortality (per 1,000)	7,498	65.63	60.81/57.47	0	571.4
birth assistance (rate)	7,123	0.428	0.304/0.279	0	1
population (per km^2^)	7,513	102.8	299.2/200.7	0	7,657
electricity (rate)	7,500	0.199	0.305/0.278	0	1
urban (rate)	7,515	0.191	0.298/0.273	0	1

Units of observation are circular zones of 2 km (5 km) radius around urban (rural) DHS clusters in panel A, and PRIO-GRID cells in panel B. Reported standard deviations are the overall standard deviation and the standard deviation within country-years.

**Fig 2:**
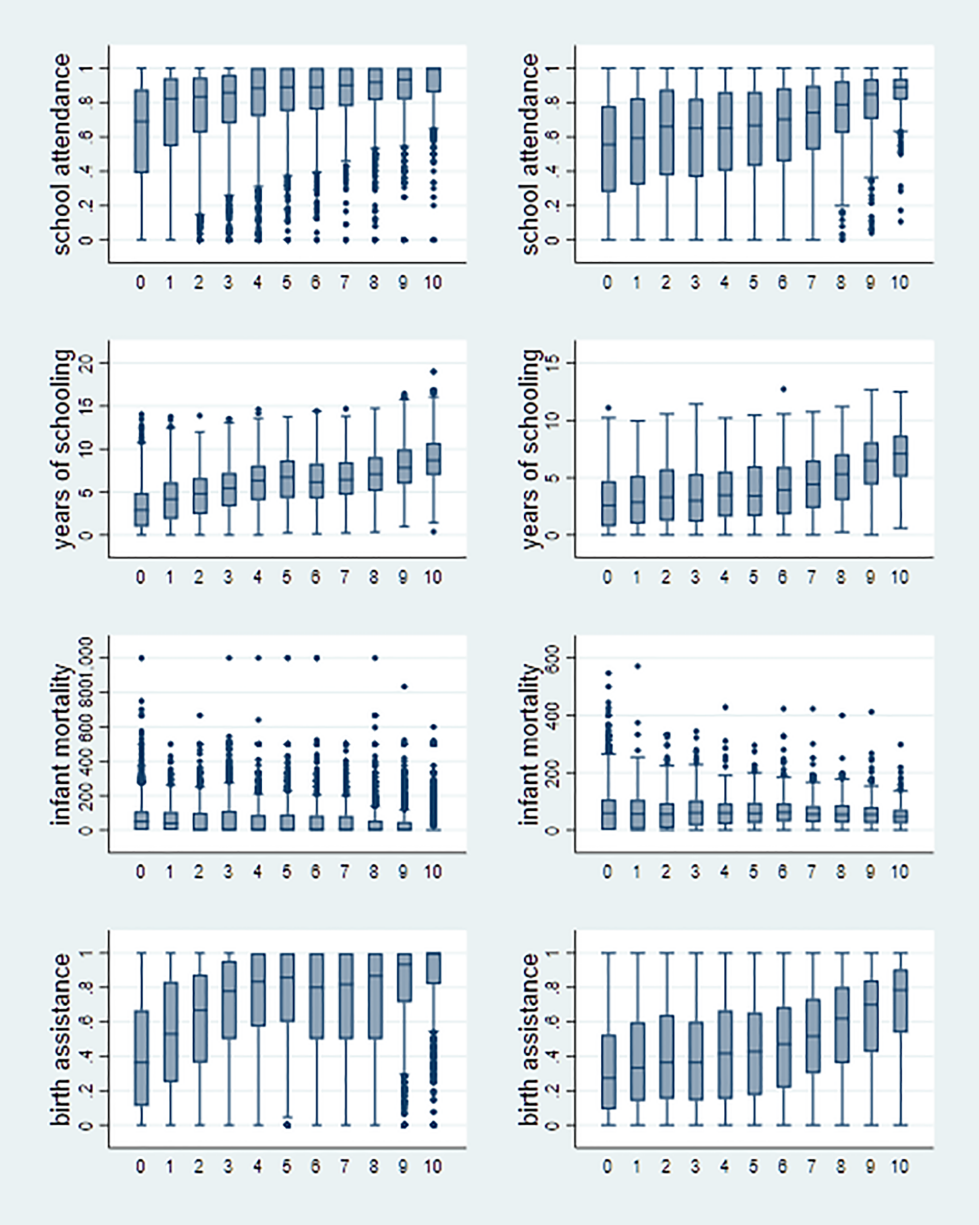
Distribution of development indicators over bins of nighttime lights. Graphs show box plots for four development indicators over 11 bins of nighttime light values. The development indicators are: primary school attendance, years of schooling, share of births assisted by professional health personnel, and infant mortality. Spatial units are circular zones of 2 km (5 km) radius around urban (rural) DHS clusters in the left column and PRIO-GRID cells in the right column. In each graph, the first bin contains all spatial units with zero average nighttime light; the remaining bins are based on the deciles of the nighttime lights distribution of the spatial units with above-zero nighttime lights. Boxes indicate the range from the 25th to the 75th percentile (interquartile range), with the median as a horizontal line; whiskers indicate the range of values within up to 1.5 times the interquartile range; and dots indicate values outside this range.

Fig 2 provides a first descriptive notion of the relationship between nighttime lights and our human development indicators related to education and health. (We omit the indicators of household wealth, as they are not comparable across countries.) It shows the distribution of each of these development indicators across 11 bins of nighttime light values. In each graph, the first bin contains all spatial units with zero nighttime lights while the remaining ten bins are based on the deciles of the nighttime lights distribution of spatial units with above-zero nighttime lights. Graphs on the left are based on the sample of circular zones, and graphs on the right on the sample of PRIO-GRID cells.

Fig 2 offers two main insights: First, human development seems to advance steadily with nighttime lights. Second, the standard deviations in our development indicators are large within each of these bins. These large standard deviations could result from, e.g., omitted variables or errors in the measurement of nighttime lights and local human development. We next turn to a regression analysis to shed further light onto the association between nighttime lights and our development indicators. We thereby address the issue of omitted variables. For a thorough discussion on the implications of measurement errors for the usefulness of nighttime lights as a proxy for economic output, see [7, 21].

### Empirical specification

Our main specification is a simple linear regression:

$$\begin{array}{c} DHS_{ict}=\alpha_{ct}+\beta \ln\left({\text{light}}_{ict}+0.01\right)+X_{\text{ict}}\Gamma +\epsilon_{ict} \end{array}$$ (1)

where *DHS*_*ict*_ represents one of our six development indicators in circular zone or PRIO-GRID cell *i* of country *c* in year *t*.

Three aspects of this specification are worth discussing. First, the country-year fixed effects *α*_*ct*_ imply that we look at the effect of nighttime lights to explain differences in our development indicators within single country-years or, equivalently, single surveys. Among others, we thereby account for differences across countries and waves in the survey items available to assess household wealth, and for changes in satellites and their sensor settings over time.

Second, we present regressions with various sets of control variables **X**_**ict**_. In many regressions, we control for population density. We could alternatively divide average nighttime light by population to get a variable with the flavor of GDP per capita. However, controlling for population density is more flexible. In some regressions, we further control for electrification and urbanization. We do so because previous studies have shown that nighttime lights contain information about the size of urban clusters [12, 32, 33] as well as about electrification at the local level [34, 35]. In these regressions, the coefficient *β* measures the additional information contained in local nighttime lights that is not yet reflected in local population density, electrification and urbanization. There is a practical and a technical reason for why we control for electrification and urbanization only in some specifications. The practical reason is that many researchers who are in need of a proxy for local human development lack information on local electrification (and urbanization). Hence, they are interested in the relation between nighttime lights and human development unconditional of electrification (and urbanization). The technical reason is that our measures of electrification and urbanization are based on the same surveyed households as our development indicators. Hence, in a horse-race between electrification and urbanization, on the one hand, and nighttime lights, on the other hand, the former may outperform the latter not because they are truly more closely related to human development, but simply because they are based on the same households as our human development indicators while nighttime lights capture human activity from all households within the entire spatial unit.

Third, we use the logarithm of nighttime lights and population density because the distributions of these variables are strongly right-skewed in both our samples. The log-transformed values give more weight to variation in nighttime lights and population density in the lower segments where observations are concentrated. Given the high share of spatial units with zero average nighttime light in our samples, we follow the previous literature [9, 10, 13, 14] in adding a constant of 0.01 before taking the logarithm in order not to drop these observations from our samples. This adjustment is in line with our assumption that zero average nighttime light does not typically reflect complete absence of light emissions. To reach a better understanding of the implications of zero average nighttime light for local human development, we also use two alternative nighttime lights variables, which are regularly used in the literature: a dummy variable that is equal to one if a spatial unit has non-zero average nighttime light, and zero otherwise; and the logarithm of average nighttime light without adding a small constant. In addition, we also use the inverse hyperbolic sine (IHS) transformation [36]:

$$\begin{array}{c} \text{IHS(light)}=\text{ln}(\text{light}+\sqrt{{\left(\text{light}\right)}^{2}+1}) \end{array}$$

This transformation allows retaining zero-valued observations, but behaves similar to a log transformation for large values of light. Intuitively, it adds a non-negligible constant if and only if necessary, i.e., if light is low.

Finally, our standard errors *ϵ*_*ict*_ are obtained by two-way clustering on countries and years.

## Results

### Main results

Tables 2–7 show how our six indicators of local human development are associated with nighttime lights within country-years. In each table, Panel A presents estimates based on our small circular zones around DHS clusters, and Panel B estimates based on PRIO-GRID cells.

**Table 2:** Nighttime lights and household wealth.

	(1)	(2)	(3)	(4)	(5)	(6)
Panel A: Small circular zones						
ln(light+0.01)	0.274*** (0.008)				0.238*** (0.010)	0.057*** (0.015)
I(light>0)		1.597*** (0.058)				
ln(light)			0.415*** (0.016)			
IHS(light)				0.571*** (0.013)		
ln(population)					0.094** (0.036)	0.067** (0.024)
electricity						1.553*** (0.109)
urban						0.761*** (0.178)
*R*^2^	0.527	0.393	0.547	0.547	0.528	0.701
Observations	26,759	26,759	12,720	26,759	25,932	25,932
Panel B: PRIO-GRID cells						
ln(light+0.01)	0.326*** (0.016)				0.346*** (0.026)	0.084*** (0.017)
I(light>0)		0.778*** (0.055)				
ln(light)			0.281*** (0.014)			
IHS(light)				0.798*** (0.131)		
ln(population)					-0.042 (0.077)	0.032 (0.048)
electricity						2.043*** (0.097)
urban						0.724*** (0.095)
*R*^2^	0.357	0.190	0.332	0.238	0.359	0.644
Observations	7,144	7,144	4,100	7,144	7,131	7,131

Dependent variable is household wealth. Linear regressions with country-year fixed effects on a sample including all geo-coded DHS in African countries from 1992-2013. Units of observation are circular zones of 2 km (5 km) radius around urban (rural) DHS clusters in panel A, and PRIO-GRID cells in panel B. Standard errors are clustered at the country level and the year level.

*** and ** indicate significance at the 1 and 5%-level, respectively.

**Table 3:** Nighttime lights and electricity-free wealth.

	(1)	(2)	(3)	(4)	(5)	(6)
Panel A: Small circular zones						
ln(light+0.01)	0.186*** (0.030)				0.188*** (0.027)	0.111*** (0.034)
I(light>0)		1.154*** (0.158)				
ln(light)			0.217*** (0.070)			
IHS(light)				0.366*** (0.072)		
ln(population)					-0.005 (0.048)	-0.013 (0.037)
electricity						1.166*** (0.285)
urban						-0.054 (0.378)
*R*^2^	0.247	0.208	0.365	0.230	0.247	0.295
Observations	26,702	26,702	12,669	26,702	25,875	25,875
Panel B: PRIO-GRID cells						
ln(light+0.01)	0.245*** (0.037)				0.268*** (0.044)	0.117*** (0.022)
I(light>0)		0.662*** (0.073)				
ln(light)			0.192*** (0.038)			
IHS(light)				0.518*** (0.164)		
ln(population)					-0.052 (0.065)	-0.009 (0.044)
electricity						1.217*** (0.391)
urban						0.387** (0.178)
*R*^2^	0.263	0.201	0.196	0.177	0.266	0.353
Observations	7,275	7,275	4,154	7,275	7,262	7,262

Dependent variable is e-free wealth. Linear regressions with country-year fixed effects on a sample including all geo-coded DHS in African countries from 1992-2013. Units of observation are circular zones of 2 km (5 km) radius around urban (rural) DHS clusters in panel A, and PRIO-GRID cells in panel B. Standard errors are clustered at the country level and the year level.

*** and ** indicate significance at the 1 and 5%-level, respectively.

**Table 4:** Nighttime lights and school attendance.

	(1)	(2)	(3)	(4)	(5)	(6)
Panel A: Small circular zones						
ln(light+0.01)	0.029*** (0.003)				0.023*** (0.003)	0.006*** (0.002)
I(light>0)		0.179*** (0.019)				
ln(light)			0.036*** (0.004)			
IHS(light)				0.059*** (0.006)		
ln(population)					0.017*** (0.005)	0.015** (0.006)
electricity						0.177*** (0.033)
urban						0.050*** (0.015)
*R*^2^	0.492	0.473	0.336	0.489	0.502	0.527
Observations	28,788	28,788	14,177	28,788	27,531	27,439
Panel B: PRIO-GRID cells						
ln(light+0.01)	0.045*** (0.006)				0.039*** (0.005)	0.007* (0.004)
I(light>0)		0.111*** (0.014)				
ln(light)			0.037*** (0.006)			
IHS(light)				0.099*** (0.027)		
ln(population)					0.014 (0.011)	0.023* (0.011)
electricity						0.252*** (0.057)
urban						0.087*** (0.020)
*R*^2^	0.515	0.486	0.496	0.488	0.517	0.559
Observations	7,436	7,436	4,308	7,436	7,423	7,411

Dependent variable is school attendance. Linear regressions with country-year fixed effects on a sample including all geo-coded DHS in African countries from 1992-2013. Units of observation are circular zones of 2 km (5 km) radius around urban (rural) DHS clusters in panel A, and PRIO-GRID cells in panel B. Standard errors are clustered at the country level and the year level.

***, ** and * indicate significance at the 1, 5 and 10%-level, respectively.

**Table 5:** Nighttime lights and years of schooling.

	(1)	(2)	(3)	(4)	(5)	(6)
Panel A: Small circular zones						
ln(light+0.01)	0.508*** (0.028)				0.425*** (0.025)	0.064* (0.033)
I(light>0)		2.853*** (0.170)				
ln(light)			0.889*** (0.070)			
IHS(light)				1.093*** (0.056)		
ln(population)					0.225*** (0.048)	0.171*** (0.039)
electricity						3.102*** (0.259)
urban						1.525*** (0.486)
*R*^2^	0.605	0.535	0.468	0.629	0.623	0.712
Observations	28,860	28,860	14,236	28,860	27,588	27,491
Panel B: PRIO-GRID cells						
ln(light+0.01)	0.591*** (0.059)				0.608*** (0.060)	0.160*** (0.045)
I(light>0)		1.263*** (0.158)				
ln(light)			0.541*** (0.061)			
IHS(light)				1.503*** (0.347)		
ln(population)					-0.033 (0.112)	0.094 (0.082)
electricity						3.576*** (0.298)
urban						1.228*** (0.297)
*R*^2^	0.627	0.552	0.581	0.591	0.627	0.726
Observations	7,442	7,442	4,309	7,442	7,429	7,416

Dependent variable is years of schooling. Linear regressions with country-year fixed effects on a sample including all geo-coded DHS in African countries from 1992-2013. Units of observation are circular zones of 2 km (5 km) radius around urban (rural) DHS clusters in panel A, and PRIO-GRID cells in panel B. Standard errors are clustered at the country level and the year level.

*** and * indicate significance at the 1 and 10%-level, respectively.

**Table 6:** Nighttime lights and infant mortality.

	(1)	(2)	(3)	(4)	(5)	(6)
Panel A: Small circular zones						
ln(light+0.01)	-1.435*** (0.312)				-1.281*** (0.301)	0.074 (0.297)
I(light>0)		-7.836*** (1.961)				
ln(light)			-2.896*** (0.579)			
IHS(light)				-3.103*** (0.619)		
ln(population)					-0.322 (0.403)	-0.145 (0.419)
electricity						-15.587*** (2.709)
urban						-2.856 (2.197)
*R*^2^	0.050	0.049	0.039	0.050	0.052	0.054
Observations	28,818	28,818	14,107	28,818	27,550	27,550
Panel B: PRIO-GRID cells						
ln(light+0.01)	-1.445** (0.576)				-2.456*** (0.564)	-0.779 (0.606)
I(light>0)		-4.262** (1.971)				
ln(light)			-1.073** (0.504)			
IHS(light)				-2.789* (1.553)		
ln(population)					2.195** (1.011)	1.720* (0.958)
electricity						-13.241** (5.326)
urban						-4.727** (2.200)
*R*^2^	0.104	0.104	0.101	0.103	0.105	0.108
Observations	7,498	7,498	4,340	7,498	7,485	7,485

Dependent variable is infant mortality. Linear regressions with country-year fixed effects on a sample including all geo-coded DHS in African countries from 1992-2013. Units of observation are circular zones of 2 km (5 km) radius around urban (rural) DHS clusters in panel A, and PRIO-GRID cells in panel B. Standard errors are clustered at the country level and the year level.

***, ** and * indicate significance at the 1, 5 and 10%-level, respectively.

**Table 7:** Nighttime lights and birth assistance.

	(1)	(2)	(3)	(4)	(5)	(6)
Panel A: Small circular zones						
ln(light+0.01)	0.053*** (0.004)				0.044*** (0.004)	0.012*** (0.003)
I(light>0)		0.311*** (0.025)				
ln(light)			0.074*** (0.006)			
IHS(light)				0.108*** (0.008)		
ln(population)					0.020*** (0.006)	0.015*** (0.005)
electricity						0.261*** (0.030)
urban						0.149*** (0.031)
*R*^2^	0.426	0.379	0.298	0.428	0.438	0.495
Observations	27,901	27,901	13,905	27,901	26,636	26,636
Panel B: PRIO-GRID cells						
ln(light+0.01)	0.061*** (0.005)				0.064*** (0.005)	0.014*** (0.004)
I(light>0)		0.141*** (0.011)				
ln(light)			0.054*** (0.006)			
IHS(light)				0.143*** (0.035)		
ln(population)					-0.006 (0.010)	0.009 (0.006)
electricity						0.379*** (0.038)
urban						0.153*** (0.025)
*R*^2^	0.459	0.402	0.462	0.421	0.460	0.553
Observations	7,123	7,123	4,139	7,123	7,110	7,110

Dependent variable is birth assistance. Linear regressions with country-year fixed effects on a sample including all geo-coded DHS in African countries from 1992-2013. Units of observation are circular zones of 2 km (5 km) radius around urban (rural) DHS clusters in panel A, and PRIO-GRID cells in panel B. Standard errors are clustered at the country level and the year level.

*** indicates significance at the 1%-level.

Each development indicator is regressed on the logarithm of average nighttime light after adding a small constant in column (1) of these tables. For all our development indicators and both types of spatial units, we find a positive and statistically highly significant association between nighttime lights and human development. The large sample sizes are the reason for the relatively small standard errors despite the relatively large standard deviations seen in Fig 2.

The point estimates of the regressions based on circular zones imply that an increase in nighttime lights by 100 percent (which corresponds to a bit less than a within country-year standard deviation increase on the sample average) is approximately associated with an increase in the mean household wealth value by 0.27, and by 0.19 for electricity-free wealth; an increase in primary school attendance by 3 in 100 children at primary school age; an increase in adults’ years of education by 0.5 years; a drop in infant mortality by 1.4 deaths per 1000 live births; and an increase in the share of births assisted by professional health personnel by 5 in 100 births. In these regressions, nighttime lights explain 53 percent of the variation in household wealth and 25 percent of the variation in electricity-free wealth; 49 percent of the variation in school attendance and 61 percent of the variation in adults’ education; and 5 percent of the variation in infant mortality and 43 percent of the variation in professional birth assistance.

The point estimates suggest stronger associations across PRIO-GRID cells. In addition, nighttime lights also explain a larger share of the variation in most human development indicators when we use PRIO-GRID cells than when we use the smaller circular zones. Hence, nighttime lights appear to be a slightly more precise measure of human development in PRIO-GRID cells than at higher spatial resolution. This finding is in line with previous studies documenting that accuracy in estimating GDP with nighttime lights is higher for more aggregate spatial resolutions [2, 7].

In column (2) of Tables 2–7, we use a dummy variable indicating whether average nighttime light is non-zero. The estimated coefficients are again statistically significant in all cases, but—not surprisingly—this dummy variable explains a smaller share in the variation in our human development indicators. In columns (3), we use the logarithm of average nighttime light without adding a small constant before the log transformation. As a result, we exclude all spatial units with zero nighttime lights. The estimated coefficients are again statistically significant in all cases. A comparison with columns (1) suggests that a smaller share of the variation in our human development indicators can be explained when dropping spatial units with zero nighttime lights than when keeping these units. Hence, ln(light+0.01), which keeps spatial units with zero nighttime lights *and* makes use of the variation across spatial units with non-zero nighttime lights, tends to perform better than these two alternative nighttime lights variables.

In column (4) of Tables 2–7, we employ the inverse hyperbolic sine transformation, which also keeps spatial units with zero nighttime lights and makes use of the variation across spatial units with non-zero nighttime lights. The estimated coefficients are again statistically significant in all cases (except Table 6, Panel B). A comparison between columns (1) and (4) of Tables 2–7 suggests that ln(light+0.01) and IHS(light) can explain similar shares of the variation in human development across the small circular zones, but that the former can explain a somewhat larger share of the variation in some human development indicators across PRIO-GRID cells. Given this pattern and the frequent use of ln(light+0.01) in the literature, we use this measure in the remaining columns.

In columns (5) of Tables 2–7, we control for the logarithm of population density. We find that the estimated coefficients on nighttime lights remain similar as in columns (1). When adding the local electrification and urbanization as control variables in columns (6), the magnitude of the estimated coefficients on nighttime lights drops substantively for all our development indicators. This drop was to be expected, not least because these controls are based on the same surveyed households as the development indicators themselves, while nighttime lights are not. Nevertheless, the coefficients on nighttime lights remain statistically significant for most indicators, except infant mortality. Hence, while part of the variation in local nighttime lights is absorbed by variation in electrification and urbanization in the DHS clusters, nighttime lights still contain considerable information about local human development that goes beyond these households’ access to electricity and urbanization. Our results also confirm that higher rates of electrification and urbanization are positively associated with human development outcomes at the local level.

We can conclude that variation in nighttime lights captures a substantial share of the variation in local human development across small spatial units of different size within countries and years.

### Robustness

In this section, we discuss five robustness tests.

In our main analysis, we have used the nighttime lights data from the newer satellite for all the years with data from two satellites. S2 Table presents results when using the nighttime lights data from the older satellites for all those years. For all development indicators (with the exception of infant mortality), the coefficient estimates, the standard errors and the explanatory power of nighttime lights are almost identical as in Tables 2–7. Hence, the choice of whether to use nighttime lights data from the newer or the older satellites should not be a main concern for social scientists, at least when including country-year (or year) fixed effects to control for changes in satellites and their sensor settings.

Our main results are based on the average nighttime lights in the spatial units. S3 Table presents results based on the sum of nighttime lights in these units instead. The pattern remains the same, but the coefficient estimates and the share of variation in human development explained by nighttime lights tend to become smaller.

We have included country-year fixed effects in our main analysis. We have thus looked at the association between nighttime lights and local human development *within* countries. We replace the country-year fixed effects by year fixed effects in S4 Table to examine whether nighttime lights are positively associated with human development when comparing subnational spatial units *across* the entire African continent. Given that the methodology used to derive our wealth indicators is specific to country and survey waves, we exclude them from this part of the analysis. We find that local nighttime lights are again positively correlated with education and health at the local level. While effect sizes are very similar to those in the within-country regressions (except for infant mortality, where the analysis across countries yields a stronger association), nighttime lights have substantially lower explanatory power in cross-country than in within-country regressions.

Next, we check whether the relationship between nighttime lights and local human development holds also when we account for the substantial variation in the numbers of observations that underlie our local development indicators. For the circular zones around DHS clusters, this variation is driven by differences in the number of households sampled per cluster. For the PRIO-GRID cells, the number of observations which feed into cell-level aggregates is also driven by the number of clusters that fall within a cell. We thus use weighted regressions, in which we apply as weights the number of observations used to compute each development indicator value for a given zone. The weighted regressions presented in S5 Table yield similar coefficient estimates as the unweighted regressions presented in Tables 2–7, except that the coefficients on nighttime lights become statistically insignificant in some regressions for PRIO-GRID cells when controlling for local electrification and urbanization. The weighted regressions however tend to have higher explanatory power than the unweighted regressions.

Finally, in our main regressions, we have treated each zone as an independent observation, no matter whether it is located in isolation or whether it is part of an agglomeration of settlements where several clusters were sampled. We now take into account whether a circular zone overlaps with other circular zones of the same survey wave. We do this by again applying weights to our main regressions based on circular zones around DHS clusters. This time we use as weights $w_{i}=\frac{1}{1+Z_{i}}$, where *Z*_*i*_ is the number of other zones from the same survey wave that overlap with a given zone *i*. Thereby we give more weight to spatial zones located in isolation, and less weight to zones located in an area where we have information from several nearby DHS clusters. Results are presented in S6 Table. They are very similar as our main results presented in Tables 2–7.

### Heterogeneity

So far, we have shown average correlations between nighttime lights and development indicators across a wide range of economic and political contexts. We now check whether the association between nighttime lights and local development differs depending on the country’s economic and political structure. For that purpose, we use three measures that capture the economic and political country context, namely GDP per capita in constant 2005 dollars [37], value added in agriculture (including forestry, hunting, fishing, cultivation of crops, and livestock production) as a share of GDP [37], and the level of democracy measured by the Polity2 score [38]. For each of these variables, we obtain the respective values for each country for each survey year. In addition, we check whether the association between nighttime lights and local development has changed over time.

**Fig 3:**
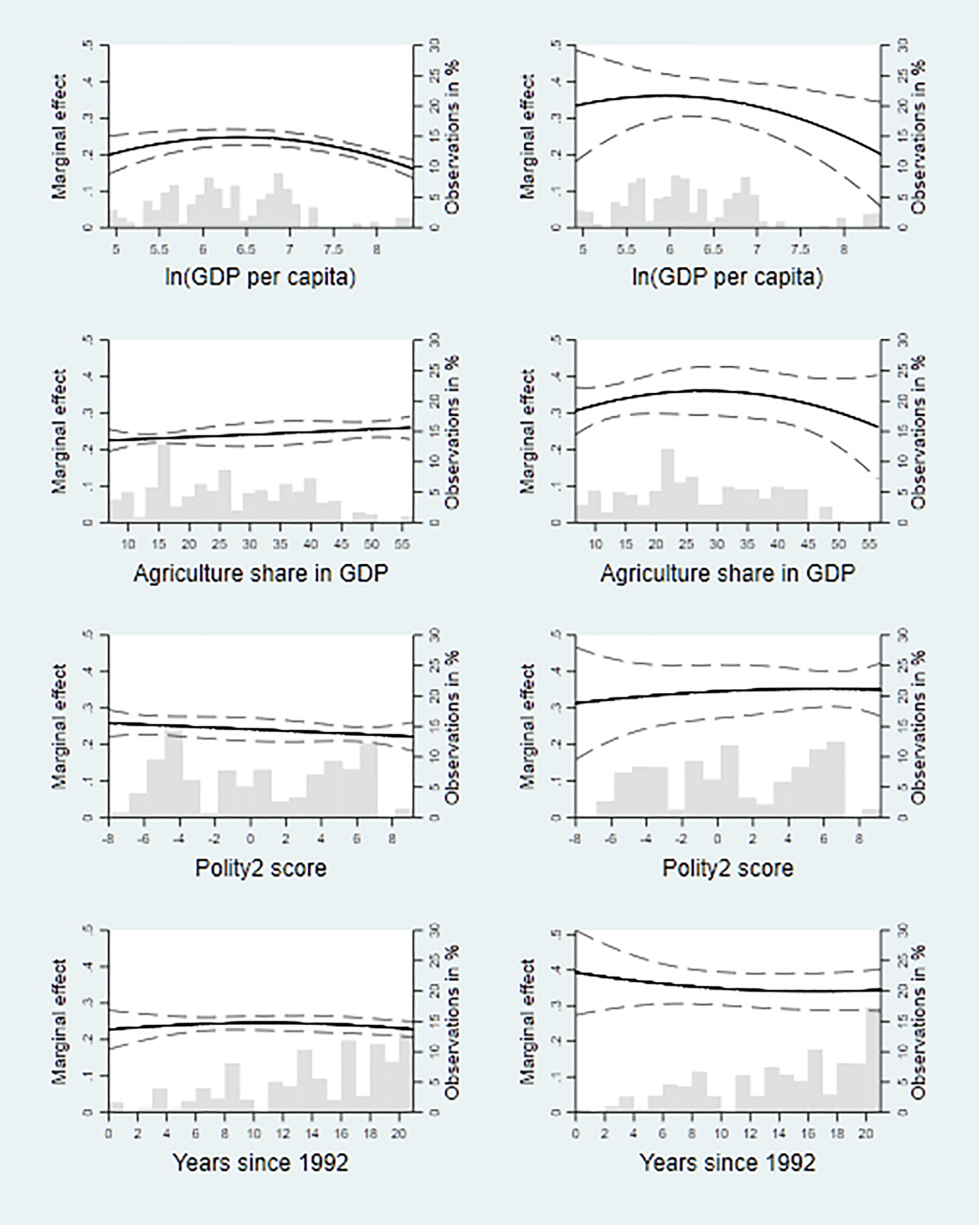
Nighttime lights and household wealth for different country characteristics and over time. Graphs show the marginal effect of ln(light+0.01) on household wealth for different values of four different modifying variables: GDP per capita (in logs), share of agricultural value added in GDP, Polity2 score, and time (measured in years since 1992). Units of observation are circular zones of 2 km (5 km) radius around urban (rural) DHS clusters in the left column and PRIO-GRID cells in the right column. In each graph, the bold curve shows the point estimate of the marginal effect and the dashed curves the corresponding 95% confidence interval (see Eqs 2 and 3). The grey bars indicate the distribution of the modifying variable.

We proceed as follow. First, we sequentially choose GDP per capita (in logs), the share of agricultural value added in GDP, the Polity2 score and time as modifying variable *Z*_*ct*_. Second, we interact *Z*_*ct*_ and its square term with our main explanatory variable ln(light_*ict*_ + 0.01). We then estimate

$$\begin{array}{c} DHS_{ict}=\alpha_{ct}+\left[\beta_{1}+\beta_{2}Z_{ct}+\beta_{3}{\left(Z_{ct}\right)}^{2}\right]\ln\left({\text{light}}_{ict}+0.01\right)+\gamma X_{ict}+\epsilon_{ict} \end{array}$$ (2)

where *DHS*_*ict*_ is household wealth (as most researchers may primarily use nighttime lights as a proxy for economic development) and *X*_*ict*_ is population density (in logs), as in column (5) of Table 2. Finally, we illustrate the marginal effect

$$\begin{array}{c} \frac{\partial DHS_{ict}}{\partial \ln({\text{light}}_{ict}+0.01)}=\beta_{1}+\beta_{2}Z_{ct}+\beta_{3}{\left(Z_{ct}\right)}^{2} \end{array}$$ (3)

and the corresponding confidence interval for the different values of the modifying variable *Z*_*ct*_ in Fig 3.

Fig 3 reveals the following patterns: First, the marginal effect of nighttime lights on household wealth is non-monotonic in GDP per capita: It tends to be increasing for low values of GDP per capita and decreasing for high values. Second, this marginal effect tends to be increasing in the share of agricultural value added in GDP for low shares, while the pattern for high shares differs across our two types of spatial units. Third, there is no clear relation between the Polity2 score and this marginal effect. Lastly, Fig 3 suggests that the marginal effect of nighttime lights on household wealth has been fairly constant over time when using the small circular areas, but slightly decreasing over time when using the PRIO-GRID cells.

## Conclusions

Nighttime lights, calculated from weather satellite recordings, are increasingly used by social scientists as a proxy for economic activity or development in regions for which disaggregated data from statistical offices are not available. This paper complements the existing evidence underpinning the use of nighttime lights in the social sciences by two important aspects: First, we have studied whether nighttime lights are an accurate proxy for development at a higher spatial resolution. We confirm that the variation in nighttime lights captures a substantial share of the variation in local development across very small spatial units, such as municipalities, as well as across PRIO-GRID cells. This finding may encourage social scientists to consider nighttime lights as a proxy for development in studies exploiting the high spatial resolution of the nighttime lights data or relying on the PRIO-GRID data.

Second, we have examined whether nighttime lights can capture not only economic development, but also other dimensions of human development at the local level. We can indeed confirm that nighttime lights capture human development as measured in household wealth, education and health. That is, nighttime lights that are relatively intense are indicative of a relatively wealthy, well-educated and healthy local population. This finding implies that future studies concerned with subnational differences in human development may well use nighttime lights in absence of other reliable disaggregated data. It also implies that some of the existing studies that used nighttime lights to study, e.g., comparative development merit broader interpretations, considering that differences in nighttime lights across subnational regions reveal not only differences in economic activity, but also differences in the level of human development of the local population.

## Supporting information

S1 Table List of countries and DHS waves in our sample. (PDF)

S2 Table Results based on nighttime lights from older satellites. (PDF)

S3 Table Results based on the sum of nighttime lights. (PDF)

S4 Table Results based on variation across African countries. (PDF)

S5 Table Results from weighted regressions based on the number of observations. (PDF)

S6 Table Results from weighted regressions based on the inverse number of overlapping circular zones. (PDF)

S1 Fig Share of spatial units with zero average nighttime light across countries and over time. (PDF)
