# Supplementary material for: Nighttime lights as a proxy for human development at the local level
Source: PLoS One. 2018 Sep 5;13(9):e0202231. doi: 10.1371/journal.pone.0202231 (PMC6124706; doi:10.1371/journal.pone.0202231)
Supplement: S1 Table — (PDF) [file pone.0202231.s001.pdf]

S1 Table: List of countries and DHS waves in our sample

County	DHS waves
Benin	1996, 2001, 2011
Burkina Faso	1998, 2003, 2010
Burundi	2010
Cameroon	2004, 2011
Cote d'Ivoire	1994, 1998, 2011
Dem. Rep. Congo	2007, 2013
Egypt	1992, 1995, 2000, 2005, 2008
Ethiopia	2000, 2005, 2010
Ghana	1998, 2003, 2008
Guinea	1999, 2005, 2012
Kenya	2003, 2008
Lesotho	2004, 2009
Liberia	2006, 2013
Madagascar	1997, 2008
Malawi	2000, 2004, 2010
Mali	1995, 2001, 2006, 2012
Mozambique	2011
Niger	1998
Nigeria	2003, 2008, 2013
Namibia	2000, 2006, 2013
Rwanda	2005, 2010
Sierra Leone	2008, 2013
Senegal	1997, 2005, 2010
Swaziland	2006
Togo	1998, 2013
Tanzania	1999, 2009
Uganda	2000, 2006, 2011
Zambia	2007, 2013
Zimbabwe	1999, 2005, 2010
