# Supplementary material for: Nighttime lights as a proxy for human development at the local level
Source: PLoS One. 2018 Sep 5;13(9):e0202231. doi: 10.1371/journal.pone.0202231 (PMC6124706; doi:10.1371/journal.pone.0202231)
Supplement: S2 Table — (PDF) [file pone.0202231.s002.pdf]

S2 Table: Results based on nighttime lights from older satellites

	(1)	(2)	(3)	(4)	(5)	(6)	(7)	(8)	(9)	(10)	(11)	(12)
Dep. var.:	household wealth		e-free wealth		school attendance		years of schooling		infant mortality		birth assistance	
Panel A: Small circular zones												
ln(light+0.01)	0.239***	0.057***	0.187***	0.109***	0.023***	0.006***	0.426***	0.062*	-1.248***	0.143	0.045***	0.012***
	(0.010)	(0.014)	(0.027)	(0.033)	(0.003)	(0.002)	(0.025)	(0.033)	(0.306)	(0.326)	(0.004)	(0.003)
ln(population)	0.094**	0.066**	-0.003	-0.012	0.017***	0.015**	0.225***	0.172***	-0.364	-0.186	0.020***	0.015**
	(0.036)	(0.024)	(0.048)	(0.037)	(0.005)	(0.006)	(0.051)	(0.041)	(0.397)	(0.409)	(0.006)	(0.005)
electricity		1.550***		1.170***		0.177***		3.107***		-15.824***		0.261***
		(0.110)		(0.290)		(0.033)		(0.265)		(2.790)		(0.030)
urban		0.761***		-0.051		0.051***		1.529***		-2.987		0.149***
		(0.177)		(0.378)		(0.015)		(0.481)		(2.205)		(0.031)
R^2	0.529	0.701	0.246	0.294	0.502	0.527	0.623	0.712	0.052	0.054	0.437	0.495
Observations	25,932	25,932	25,875	25,875	27,531	27,439	27,588	27,491	27,550	27,550	26,636	26,636
Panel B: PRIO-GRID cells												
ln(light+0.01)	0.347***	0.084***	0.268***	0.116***	0.039***	0.007*	0.609***	0.156***	-2.556***	-0.859	0.064***	0.013***
	(0.026)	(0.016)	(0.043)	(0.022)	(0.005)	(0.004)	(0.061)	(0.045)	(0.557)	(0.671)	(0.005)	(0.004)
ln(population)	-0.038	0.034	-0.048	-0.007	0.014	0.023*	-0.024	0.100	2.265**	1.798*	-0.005	0.009
	(0.078)	(0.048)	(0.065)	(0.044)	(0.011)	(0.011)	(0.117)	(0.087)	(1.023)	(0.978)	(0.010)	(0.006)
electricity		2.040***		1.223***		0.252***		3.578***		-13.247**		0.378***
		(0.098)		(0.395)		(0.057)		(0.297)		(5.423)		(0.037)
urban		0.724***		0.386**		0.087***		1.236***		-4.788*		0.153***
		(0.095)		(0.179)		(0.020)		(0.295)		(2.386)		(0.025)
R^2	0.360	0.644	0.265	0.352	0.518	0.560	0.627	0.726	0.106	0.108	0.460	0.553
Observations	7,131	7,131	7,262	7,262	7,423	7,411	7,429	7,416	7,485	7,485	7,110	7,110

Notes: Linear regressions with country-year fixed effects on a sample including all geo-coded DHS in African countries from 1992-2013. Unlike in the main analysis, nighttime lights data from the older satellites are used for all years for which data are available from two different satellites. Units of observation are circular zones of 2 km (5 km) radius around urban (rural) DHS clusters in panel A, and PRIO-GRID cells in panel B. All variables are described in the main text. Standard errors are clustered at the country level and the year level. ***, **, * indicate significance at the 1, 5 and 10%-level, respectively.
