# Supplementary material for: Nighttime lights as a proxy for human development at the local level
Source: PLoS One. 2018 Sep 5;13(9):e0202231. doi: 10.1371/journal.pone.0202231 (PMC6124706; doi:10.1371/journal.pone.0202231)
Supplement: S3 Table — (PDF) [file pone.0202231.s003.pdf]

S3 Table: Results based on the sum of nighttime lights

	(1)	(2)	(3)	(4)	(5)	(6)	(7)	(8)	(9)	(10)	(11)	(12)
Dep. var.:	household wealth		e-free wealth		school attendance		years of schooling		infant mortality		birth assistance	
Panel A: Small circular zones												
ln(sum of light)	0.159***	0.035***	0.107***	0.043***	0.019***	0.004***	0.279***	0.042***	-0.675***	-0.045	0.031***	0.008***
	(0.006)	(0.005)	(0.019)	(0.010)	(0.002)	(0.001)	(0.021)	(0.011)	(0.208)	(0.175)	(0.002)	(0.002)
ln(population)		0.072**		0.030		0.021***		0.192***		0.328		0.020***
		(0.029)		(0.031)		(0.007)		(0.059)		(0.502)		(0.006)
electricity		1.715***		1.029***		0.182***		3.279***		-13.774***		0.291***
		(0.086)		(0.291)		(0.038)		(0.220)		(3.863)		(0.028)
urban		0.702***		0.286		0.060***		1.284***		-1.876		0.150***
		(0.096)		(0.199)		(0.018)		(0.291)		(2.051)		(0.025)
R^2	0.376	0.663	0.191	0.277	0.480	0.529	0.574	0.713	0.047	0.051	0.375	0.479
Observations	26,758	25,932	26,701	25,875	28,787	27,439	28,859	27,491	28,817	27,550	27,900	26,636
Panel B: PRIO-GRID cells												
ln(sum of light)	0.080***	0.024***	0.070***	0.037***	0.010***	0.003***	0.130***	0.030***	-0.595***	-0.229	0.014***	0.003***
	(0.007)	(0.003)	(0.009)	(0.005)	(0.001)	(0.001)	(0.015)	(0.007)	(0.186)	(0.144)	(0.001)	(0.001)
ln(population)	0.091	0.052	0.034	0.011	0.027**	0.023**	0.219*	0.154*	1.310	1.551	0.020*	0.013**
	(0.075)	(0.042)	(0.060)	(0.040)	(0.011)	(0.011)	(0.121)	(0.084)	(0.990)	(1.003)	(0.011)	(0.006)
electricity		2.121***		1.306***		0.254***		3.785***		-13.899***		0.394***
		(0.097)		(0.387)		(0.060)		(0.311)		(4.590)		(0.037)
urban		0.735***		0.388**		0.084***		1.304***		-4.803**		0.157***
		(0.086)		(0.176)		(0.020)		(0.289)		(2.008)		(0.024)
R^2	0.276	0.645	0.240	0.359	0.510	0.560	0.590	0.724	0.105	0.108	0.432	0.553
Observations	7,131	7,131	7,262	7,262	7,423	7,411	7,429	7,416	7,485	7,485	7,110	7,110

Notes: Linear regressions with country-year fixed effects on a sample including all geo-coded DHS in African countries from 1992-2013. Unlike in the main analysis, we use the sum of the DN of all nighttime light pixels in a given unit (labelled sum of light). Units of observation are circular zones of 2 km (5 km) radius around urban (rural) DHS clusters in panel A, and PRIO-GRID cells in panel B. All variables are described in the main text. Standard errors are clustered at the country level and the year level. ***, **, * indicate significance at the 1, 5 and 10%-level, respectively.
