# Supplementary material for: Nighttime lights as a proxy for human development at the local level
Source: PLoS One. 2018 Sep 5;13(9):e0202231. doi: 10.1371/journal.pone.0202231 (PMC6124706; doi:10.1371/journal.pone.0202231)
Supplement: S4 Table — (PDF) [file pone.0202231.s004.pdf]

S4 Table: Results based on variation across African countries

	(1)	(2)	(3)	(4)	(5)	(6)	(7)	(8)
Dep. var.:	school attendance		years of schooling		infant mortality		birth assistance	
Panel A: Small circular zones								
ln(light+0.01)	0.025***	0.007*	0.470***	0.102*	-2.847***	-1.088**	0.048***	0.017**
	(0.005)	(0.004)	(0.046)	(0.052)	(0.701)	(0.498)	(0.006)	(0.006)
ln(population)	0.021**	0.021**	0.161	0.145	0.559	0.541	0.008	0.007
	(0.008)	(0.008)	(0.114)	(0.106)	(1.050)	(1.048)	(0.015)	(0.015)
electricity		0.174***		2.863***		-23.630***		0.214***
		(0.026)		(0.235)		(4.826)		(0.033)
urban		0.027**		1.468***		4.184***		0.150***
		(0.011)		(0.492)		(1.388)		(0.029)
R^2	0.288	0.310	0.438	0.523	0.028	0.033	0.278	0.330
Observations	27,531	27,439	27,588	27,491	27,550	27,550	26,636	26,636
Panel B: PRIO-GRID cells								
ln(light+0.01)	0.056***	0.022**	0.741***	0.277***	-4.888***	-2.933***	0.079***	0.026**
	(0.009)	(0.009)	(0.069)	(0.077)	(1.017)	(0.834)	(0.007)	(0.010)
ln(population)	-0.012	0.000	-0.289**	-0.125	5.657***	4.822***	-0.046**	-0.027
	(0.011)	(0.011)	(0.136)	(0.120)	(1.093)	(1.096)	(0.021)	(0.019)
electricity		0.237***		2.986***		-17.120***		0.327***
		(0.049)		(0.392)		(4.893)		(0.054)
urban		0.062*		1.211***		1.658		0.163***
		(0.034)		(0.419)		(4.042)		(0.025)
R^2	0.274	0.311	0.395	0.480	0.069	0.072	0.241	0.330
Observations	7,423	7,411	7,429	7,416	7,485	7,485	7,110	7,110

Notes: Linear regressions with year fixed effects on a sample including all geo-coded DHS in African countries from 1992-2013. Units of observation are circular zones of 2 km (5 km) radius around urban (rural) DHS clusters in panel A, and PRIO-GRID cells in panel B. All variables are described in the main text. Standard errors are clustered at the country level and the year level. ***, **, * indicate significance at the 1, 5 and 10%-level, respectively.
