# Supplementary material for: Nighttime lights as a proxy for human development at the local level
Source: PLoS One. 2018 Sep 5;13(9):e0202231. doi: 10.1371/journal.pone.0202231 (PMC6124706; doi:10.1371/journal.pone.0202231)
Supplement: S5 Table — (PDF) [file pone.0202231.s005.pdf]

S5 Table: Results from weighted regressions based on the number of observations

	(1)	(2)	(3)	(4)	(5)	(6)	(7)	(8)	(9)	(10)	(11)	(12)
Dep. var.:	household wealth		e-free wealth		school attendance		years of schooling		infant mortality		birth assistance	
Panel A: Small circular zones												
ln(light+0.01)	0.237***	0.058***	0.184***	0.085***	0.023***	0.004*	0.429***	0.075**	-1.320***	0.078	0.045***	0.014***
	(0.009)	(0.013)	(0.020)	(0.021)	(0.003)	(0.002)	(0.027)	(0.031)	(0.248)	(0.181)	(0.004)	(0.003)
ln(population)	0.087**	0.058**	0.036	0.020	0.020***	0.017***	0.233***	0.170***	-0.379	-0.128	0.025***	0.019***
	(0.032)	(0.021)	(0.034)	(0.029)	(0.006)	(0.006)	(0.048)	(0.039)	(0.366)	(0.369)	(0.006)	(0.005)
electricity		1.581***		1.136***		0.211***		3.086***		-15.199***		0.268***
		(0.095)		(0.172)		(0.032)		(0.247)		(2.446)		(0.027)
urban		0.742***		0.212		0.060***		1.452***		-4.647**		0.151***
		(0.150)		(0.233)		(0.019)		(0.410)		(1.854)		(0.025)
R^2	0.532	0.712	0.333	0.403	0.504	0.536	0.637	0.721	0.066	0.068	0.458	0.514
Observations	25,932	25,932	25,875	25,875	27,531	27,439	27,588	27,491	27,541	27,541	26,636	26,636
Panel B: PRIO-GRID cells												
ln(light+0.01)	0.419***	0.066	0.394***	0.225***	0.046***	0.009*	0.766***	0.057	-2.415***	-0.023	0.075***	0.011
	(0.023)	(0.039)	(0.053)	(0.069)	(0.007)	(0.005)	(0.058)	(0.074)	(0.743)	(0.752)	(0.007)	(0.007)
ln(population)	-0.059	0.028	-0.197*	-0.148*	-0.001	0.005	0.033	0.161***	0.117	-0.172	0.001	0.008*
	(0.047)	(0.032)	(0.097)	(0.075)	(0.008)	(0.006)	(0.051)	(0.039)	(0.685)	(0.725)	(0.007)	(0.004)
electricity		1.322***		1.781***		0.179***		2.806***		-6.113		0.249***
		(0.238)		(0.435)		(0.048)		(0.537)		(6.860)		(0.055)
urban		1.369***		-0.478		0.129**		2.863***		-13.697***		0.304***
		(0.372)		(0.700)		(0.046)		(0.689)		(4.179)		(0.069)
R^2	0.676	0.859	0.536	0.603	0.636	0.682	0.742	0.863	0.266	0.275	0.624	0.725
Observations	7,131	7,131	7,262	7,262	7,423	7,411	7,429	7,416	7,485	7,485	7,110	7,110

Notes: Weighted linear regressions with country-year fixed effects on a sample including all geo-coded DHS in African countries from 1992-2013. Weights are number of observations from the DHS data from which local development indicators are computed for each spatial unit. Units of observation are circular zones of 2 km (5 km) radius around urban (rural) DHS clusters in panel A, and PRIO-GRID cells in panel B. All variables are described in the main text. Standard errors are clustered at the country level and the year level. ***, **, * indicate significance at the 1, 5 and 10%-level, respectively.
