# Supplementary material for: Nighttime lights as a proxy for human development at the local level
Source: PLoS One. 2018 Sep 5;13(9):e0202231. doi: 10.1371/journal.pone.0202231 (PMC6124706; doi:10.1371/journal.pone.0202231)
Supplement: S6 Table — (PDF) [file pone.0202231.s006.pdf]

S6 Table: Results from weighted regressions based on the inverse number of overlapping circular zones

	(1)	(2)	(3)	(4)	(5)	(6)	(7)	(8)	(9)	(10)	(11)	(12)
Dep. var.:	household wealth		e-free wealth		school attendance		years of schooling		infant mortality		birth assistance	
	Small circular zones											
ln(light+0.01)	0.242***	0.062***	0.164***	0.071***	0.023***	0.004***	0.422***	0.085***	-1.181***	-0.039	0.045***	0.012***
	(0.010)	(0.008)	(0.032)	(0.016)	(0.003)	(0.001)	(0.030)	(0.021)	(0.354)	(0.295)	(0.004)	(0.003)
ln(population)	0.076	0.066**	0.029	0.025	0.023***	0.022***	0.194**	0.178***	0.269	0.312	0.022***	0.020***
	(0.045)	(0.029)	(0.039)	(0.031)	(0.007)	(0.008)	(0.070)	(0.059)	(0.489)	(0.521)	(0.007)	(0.006)
electricity		1.689***		1.011***		0.188***		3.214***		-13.865***		0.291***
		(0.085)		(0.283)		(0.037)		(0.216)		(3.736)		(0.029)
urban		0.643***		0.223		0.059***		1.191***		-1.878		0.140***
		(0.101)		(0.205)		(0.018)		(0.300)		(2.234)		(0.024)
R^2	0.466	0.663	0.221	0.275	0.501	0.528	0.627	0.713	0.049	0.051	0.414	0.478
Observations	25,932	25,932	25,875	25,875	27,531	27,439	27,588	27,491	27,550	27,550	26,636	26,636

Notes: Weighted linear regressions with country-year fixed effects on a sample including all geo-coded DHS in African countries from 1992-2013. Units of observation are circular zones of 2 km (5 km) radius around urban (rural) DHS clusters, and weights are the inverse number of overlaps with other circular zones. All variables are described in the main text. Standard errors are clustered at the country level and the year level. ***, **, * indicate significance at the 1, 5 and 10%-level, respectively.
