# Supplementary material for: Nighttime lights as a proxy for human development at the local level
Source: PLoS One. 2018 Sep 5;13(9):e0202231. doi: 10.1371/journal.pone.0202231 (PMC6124706; doi:10.1371/journal.pone.0202231)
Supplement: S1 Fig — (PDF) [file pone.0202231.s007.pdf]

S1 Fig: Share of spatial units with zero average nighttime light across countries and over time

(a) Small circular zones

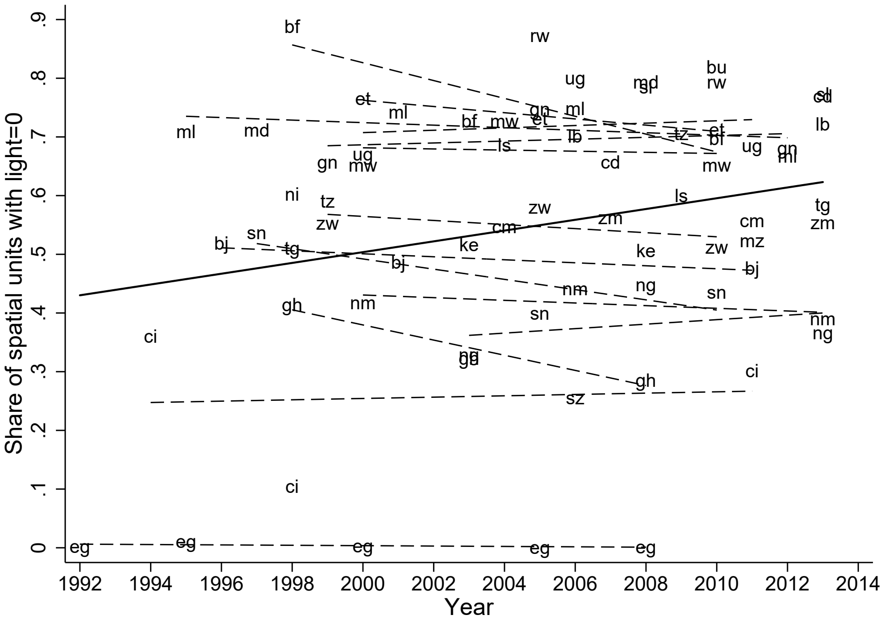

(b) PRIO-GRID cells

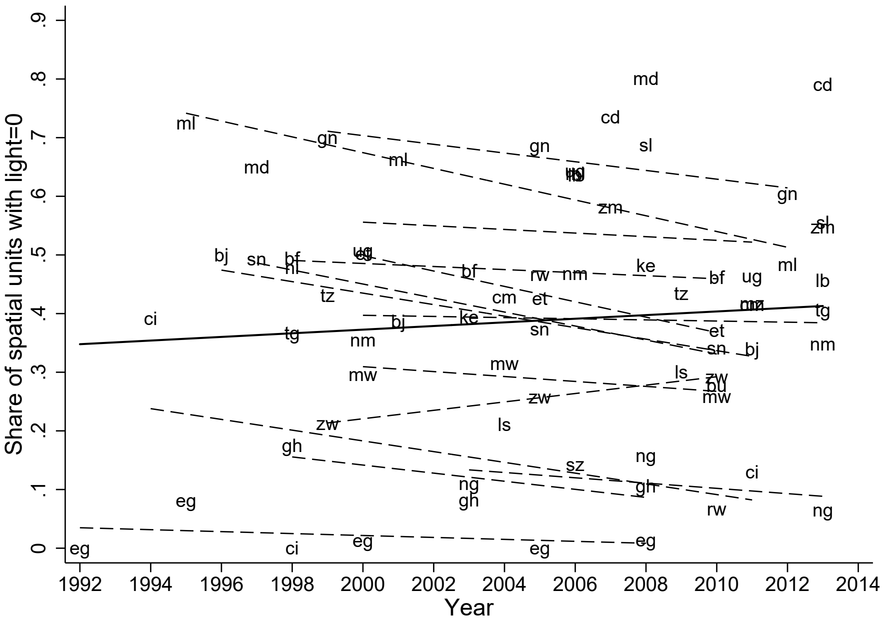

Notes: Graphs show the share of spatial units with zero average nighttime light for all country-years in which a DHS was conducted. Countries are indicated by two-letter codes, and years are on the horizontal axis. Units of observation are circular zones of 2 km (5 km) radius around urban (rural) DHS clusters in row (a), and PRIO-GRID cells in row (b). In each graph, the bold line indicates the best linear fit between the year and the share of spatial units with zero average nighttime light based on all country-years in which a DHS was conducted. Each dashed line indicates the best linear fit for one of the countries with at least three DHS (see S1 Table). For example, in each graph, the dashed line closest to the horizontal axis shows the best linear fit for the five years in which a DHS was conducted in Egypt, represented by the two-letter codes "eg."
